# Genetic variation in complement regulators and susceptibility to age-related macular degeneration

**DOI:** 10.1016/j.imbio.2011.09.002

**Published:** 2012-02

**Authors:** Valentina Cipriani, Baljinder K. Matharu, Jane C. Khan, Humma Shahid, Chloe M. Stanton, Caroline Hayward, Alan F. Wright, Catey Bunce, David G. Clayton, Anthony T. Moore, John R.W. Yates

**Affiliations:** a Institute of Ophthalmology, University College London, UK; b Moorfields Eye Hospital, London, UK; c Department of Medical Genetics, Cambridge Institute for Medical Research, University of Cambridge, UK; d Medical Research Council Human Genetics Unit, Institute of Genetics and Molecular Medicine, Edinburgh, UK

**Keywords:** AMD, age-related macular degeneration, ARM, age-related maculopathy, CFB, complement factor B, CFH, complement factor H, CI, confidence interval, CPI, complement factor I, CFP, complement factor P, CNV, choroidal neovascularisation, DAF, decay accelerating factor, DNA, deoxyribonucleic acid, GA, geographic atrophy, HWE, Hardy–Weinberg equilibrium, MAC, membrane attack complex, MAF, minor allele frequency, MCP, membrane cofactor protein, OR, odds ratio, RPE, retinal pigment epithelium, SNP, single nucleotide polymorphism, Age-related macular degeneration, Complement, Complement regulators, Genetic association, Genetic variation, Single nucleotide polymorphism

## Abstract

**Objectives:**

Age-related macular degeneration (AMD) is the commonest cause of blindness in Western populations. Risk is influenced by age, genetic and environmental factors. Complement activation appears to be important in the pathogenesis and associations have been found between AMD and genetic variations in complement regulators such as complement factor H. We therefore investigated other complement regulators for association with AMD.

**Methods:**

We carried out a case–control study to test for association between AMD and single nucleotide polymorphisms (SNPs) spanning the genes encoding complement factor P (CFP, properdin), CD46 (membrane cofactor protein, MCP), CD55 (decay accelerating factor, DAF) and CD59 (protectin). All cases and controls were examined by an ophthalmologist and had independent grading of fundus photographs to confirm their disease status.

**Results:**

20 SNPs were genotyped in 446 cases and 262 controls. For two SNPs with *p*-values approaching significance additional subjects were genotyped to increase the numbers to 622 cases and 359 controls. There was no evidence of association between AMD and any of the SNPs typed in CFP, CD46, CD55 or CD59.

**Conclusions:**

In a case–control sample that has shown the well established associations between AMD and variants in CFH, CFB and C3 there was absence of association with SNPs in CFP, CD46, CD55 and CD59. This suggests that these are not important susceptibility genes for AMD.

## Introduction

Age-related macular degeneration (AMD) is a major cause of visual impairment in people over 50 years of age and the commonest cause of blindness in Western populations (Jager et al. 2008). It affects the macula, the region of the retina rich in photoreceptors which provides detailed central vision. In the early stages of the disease (age-related maculopathy, ARM), deposits called drusen form between the retinal pigment epithelium (RPE) and underlying choroid. Later in the disease there is atrophy of the RPE and overlying photoreceptor cells (geographic atrophy, GA) and/or aberrant choroidal neovascularisation (CNV, also called wet AMD) (Jager et al. 2008). Susceptibility to AMD is influenced by age, ethnic background, genetic and environmental factors, particularly smoking (Jager et al. 2008; Swaroop et al. 2009).

Common variants in a number of genes have been shown to influence the risk of developing AMD (Swaroop et al. 2009), including complement factor H (CFH) (Klein et al. 2005; Haines et al. 2005; Edwards et al. 2005; Hughes et al. 2006), complement factor B (CFB) and/or complement 2 (C2) (Gold et al. 2006), complement 3 (C3) (Yates et al. 2007) and complement factor I (CFI) (Fagerness et al. 2009). The involvement of genes in the complement pathway, particularly complement regulators, together with the finding that drusen contain proteins associated with inflammation and immune-mediated processes (Mullins et al. 2000; Anderson et al. 2010) supports the hypothesis that complement activation is important in the pathogenesis of AMD. We have investigated variants in the major genes encoding proteins in the alternative complement pathway for evidence of association with AMD and the results for CFH, CFB, CFI, C3 and C5 have been reported elsewhere (Sepp et al. 2006; Yates et al. 2007; Cipriani et al. 2011). Because complement activation appears to be central to the pathogenesis of AMD, we have studied other regulators of the alternative complement pathway and the results are presented here.

## Materials and methods

### Patients and controls

The sample comprised cases with predominantly advanced AMD (GA or CNV) and spouse controls recruited from hospital ophthalmic clinics in London and the South East of England. All subjects described themselves as “white” on a recruitment questionnaire. The study had Research Ethics Committee approval and written consent was obtained from all participants. Subjects were examined by an ophthalmologist and health, lifestyle and smoking data were collected. All subjects had colour, stereoscopic fundus photography of the macular region. These images were independently graded at the Reading Centre, Moorfields Eye Hospital, London using the International Classification of Age-related Maculopathy and Macular Degeneration (Bird et al. 1995).

### SNP selection and genotyping

Genomic DNA was extracted from peripheral blood leucocytes and typed for variants spanning the genes encoding complement factor P (CFP, properdin), CD46 (membrane cofactor protein, MCP), CD55 (decay accelerating factor, DAF) and CD59 (protectin). SNPs were selected from the International HapMap Project (The International HapMap Consortium 2003) database (release 19) for the CEPH population (Utah residents with ancestry from northern and western Europe). In an initial round of genotyping, SNPs with a minor allele frequency of at least 10% were selected to cover the main blocks of linkage disequilibrium. Subsequently, genetic coverage of the four genes of interest was formally calculated using the Tagger tag SNP selection algorithm (de Bakker et al. 2005) implemented in Haploview v4.1 (Barrett et al. 2006) and reported as percentage number of CEPH HapMap SNPs (genotype rate ≥ 90%; minor allele frequency (MAF) ≥ 1%; Hardy–Weinberg equilibrium (HWE) *p*-value ≥ 10^−4^; maximum number of Mendelian errors = 1) captured by (at least) one genotyped SNP (at *r*^2^ ≥ 0.80). On the basis of this analysis, additional genotyping was carried out and the final coverage achieved was 83% for CFP, 71% for CD46, 92% for CD55 and 71% for CD59. SNPs were genotyped using the ABI PRISM SNaPshot ddNTP Primer Extension Kit and a 3100 Genetic Analyser (Applied Biosystems) with the exception of rs7060246 which was typed using Taqman (Applied Biosystems). Manufacturers’ protocols were followed.

### Statistical analysis

Differences in the demographic characteristics of cases and controls were assessed using the Fisher's Exact test for categorical variables and the two sample Mann–Whitney test for continuous variables as implemented in STATA (Version 11.1, StataCorp LP, College Station, TX). A difference was considered significant if the *p*-value was found to be less than 0.05. Genetic association analysis was conducted using PLINK v1.07 (Purcell et al. 2007). Departure from HWE was assessed in controls. SNP association analysis was performed using the Cochran–Armitage trend test and corresponding *p*-values are reported. Odds ratios (ORs) were calculated using referent minor allele and are presented with 95% confidence intervals (CIs). We performed logistic regression analyses adjusting for age and pack years of cigarette smoking to address the possibility that SNP genotypes were confounded by these demographic factors.

## Results

20 SNPs spanning the four genes of interest were genotyped in 446 cases and 262 controls. For two SNPs with *p*-values approaching statistical significance, additional subjects were genotyped to increase the numbers to 622 cases and 359 controls. Data on disease status, sex, age and smoking history of subjects are given in Table 1. The SNPs that were typed in each gene are listed in Table 2 together with the results of genotyping and tests for association. For all SNPs no departure from HWE was observed in control samples. There was no evidence of association between AMD and any of the SNPs in CFP, CD46, CD55 and CD59. Adjusting the analysis for age and pack years of cigarette smoking, excluding cases with ARM or confining the analysis to cases with either CNV or GA did not significantly alter the estimates (results not shown).

## Discussion

The complement system mediates host defence against pathogens, the elimination of immune complexes and apoptotic cells, and it facilitates adaptive immune responses (Walport 2001). There is good evidence that complement activation is important in the pathogenesis of AMD (see accompanying review, Khandhadia et al. 2012). Drusen, the hallmark lesion of early AMD, contain proteins from the alternative complement pathway including CFH, C3 and its activation products, and terminal pathway components including C5 and the membrane attack complex (MAC) (Mullins et al. 2000; Anderson et al. 2010). Patients with AMD have elevated levels of complement activation products in their circulation (Scholl et al. 2008; Reynolds et al. 2009; Hecker et al. 2010).

Complement activation is controlled by a large number of soluble and membrane bound regulatory proteins (Zipfel and Skerka 2009). CFH is the most important fluid phase regulator, present at high concentration in the plasma and body fluids and a key regulator of the alternative complement pathway. There is conclusive evidence that the common expressed variant Y402H (rs1061170) in the CFH gene influences susceptibility to AMD (Klein et al. 2005; Haines et al. 2005; Edwards et al. 2005) with heterozygotes and homozygotes for the 402H allele being 2.5 and 6 times more likely to have AMD respectively (Thakkinstian et al. 2006). Other CFH variants are independently associated with AMD (Li et al. 2006). A deletion of the neighbouring CFHR3 and CFHR1 genes has been shown to be protective for AMD (Hughes et al. 2006) and these proteins are also involved in complement regulation. CFI is a cofactor with CFH for the inactivation of C3b and SNPs at the CFI locus also show an association with AMD (Fagerness et al. 2009). In the present study, we investigated the fluid phase protein, complement factor P (properdin). CFP is a stabilising component of the alternative pathway convertases and can bind to cells and pathogens, promoting convertase assembly and targeted phagocytosis (Kemper and Hourcade 2008). We found no evidence of an association between the SNPs typed in CFP and AMD. A recently published study of genetic variants in CFP from Finland was also negative (Seitsonen et al. 2010).

Cell surfaces are also protected from complement activation by membrane bound proteins and we have investigated SNPs in the genes encoding three of these for association with AMD: CD46 (membrane cofactor protein, MCP), CD55 (decay accelerating factor, DAF) and CD59 (protectin). CD46 acts as cofactor for CFI mediated cleavage of C3b (Kim and Song 2006). CD55 inhibits the formation and accelerates the decay of C3 and C5 convertases (Kim and Song 2006). CD59 prevents final assembly of the MAC (Kimberley et al. 2007). We found no evidence of association with the SNPs typed in any of these genes. However, based on the confidence intervals for our odds ratios there is still the possibility that an association might exist up to a maximum OR of 1.8 in the case of rs7060246, and more generally in a range between 0.5 and 1.5 for other SNPs. Moreover, we cannot exclude the possibility of association with a common variant in poor LD with the SNPs typed, or indeed a rare variant that influences susceptibility to AMD.

We have studied these four complement pathway genes in a large well phenotyped case–control sample. The subjects used in the study were all examined by an ophthalmologist and had independent grading of their fundus photographs to confirm their disease status. This same sample has been used in other studies of AMD susceptibility and shows the expected associations with CFH, CFB/C2 and C3 (Sepp et al. 2006; Yates et al. 2007). The absence of association with SNPs in CFP, CD46, CD55 and CD59 suggests that these are not important susceptibility genes for AMD.

## Figures and Tables

**Table 1:** Disease status, sex, age and smoking history of subjects.^a^

	Core sample		Enlarged sample	
	Controls	Cases	Controls	Cases
Number of subjects	262	446	359	622
Disease status				
Age-related maculopathy (ARM)		19		26
Geographic atrophy (GA)		88		126
Choroidal neovascularisation (CNV)		267		373
Both GA and CNV		72		97
Sex				
Male	105 (40%)	207 (46%)	146 (41%)	283 (46%)
Female	157 (60%)	239 (54%)	213 (59%)	339 (54%)
Age				
Mean age, years (SD)	**75.7 (7.8)**	**80.4 (6.8)**	**75.1 (7.9)**	**79.4 (7.1)**
Pack years of cigarette smoking^b^				
0	**106 (41%)**	**169 (38%)**	**150 (42%)**	**230 (37%)**
0.1–20	**103 (39%)**	**127 (29%)**	**133 (37%)**	**175 (28%)**
20.1–40	**39 (15%)**	**99 (22%)**	**56 (15%)**	**137 (22%)**
>40	**14 (5%)**	**51 (11%)**	**20 (6%)**	**79 (13%)**

a Significant differences (at *p*-value ≤ 0.05) between cases and controls are reported in bold.

b Information on smoking was missing for 1 case in the enlarged sample.

**Table 2:** Association results for the 20 SNPs in the four complement regulator genes.

Gene	SNP	Minor allele (a)	Major allele (A)	Genotype counts^a^ (aa/Aa/AA)		Minor allele frequency		OR	95% CI	*p*-value^b^
				Cases	Controls	Cases	Controls			
CFP	rs909523	T	G	18/89/129	13/58/81	0.27	0.25	1.1	0.8–1.4	0.73
	rs7060246	T	C	1/30/204	1/19/137	0.08	0.07	1.1	0.7–1.8	0.95

CD46 (MCP)	rs2796267	G	A	97/205/144	43/139/80	0.45	0.43	1.1	0.9–1.3	0.52
	rs2724385	T	A	99/224/115	63/120/74	0.48	0.48	1.0	0.8–1.3	0.91
	rs2796269	T	C	55/188/200	32/109/119	0.34	0.33	1.0	0.8–1.3	0.89
	rs2724374	C	A	15/166/259	12/104/145	0.22	0.25	0.9	0.7–1.1	0.31
	rs3109808	C	A	70/231/142	48/127/84	0.42	0.43	1.0	0.8–1.2	0.66
	rs6657476	T	G	15/166/263	12/105/143	0.22	0.25	0.9	0.7–1.1	0.22
	rs7144	G	A	69/230/144	46/132/83	0.42	0.43	0.9	0.8–1.2	0.60

CD55 (DAF)	rs4844591	G	A	55/178/195	25/112/120	0.34	0.32	1.1	0.9–1.4	0.43
	rs7544288	A	G	104/272/243	66/168/124	0.39	0.42	0.9	0.7–1.1	0.19

CD59	rs1047581	C	T	39/189/203	28/94/121	0.31	0.31	1.0	0.8–1.3	0.97
	rs7046	A	G	61/195/161	37/103/110	0.38	0.35	1.1	0.9–1.4	0.35
	rs17760306	G	T	14/105/307	8/67/174	0.16	0.17	0.9	0.7–1.2	0.62
	rs831631	C	G	66/210/163	39/102/114	0.39	0.35	1.2	0.9–1.5	0.18
	rs12272807	T	C	2/72/371	2/52/205	0.09	0.11	0.8	0.5–1.1	0.15
	rs1738548	C	T	47/179/203	28/100/123	0.32	0.31	1.0	0.8–1.3	0.78
	rs2231454	A	G	3/88/528	5/59/294	0.08	0.10	0.8	0.6–1.1	0.12
	rs3181274	A	G	61/193/175	37/106/107	0.37	0.36	1.0	0.8–1.3	0.80
	rs17760694	C	T	16/131/291	14/83/163	0.19	0.21	0.8	0.6–1.1	0.22

a SNPs were genotyped in 446 cases and 262 controls. rs7544288 and rs2231454 were typed in the enlarged sample of 622 cases and 359 controls.

b *p*-value for the Cochran–Armitage trend test.
